# Antibiotic Utilization Among People With Multiple Sclerosis in the Netherlands, 2018–2020

**DOI:** 10.1002/pds.70070

**Published:** 2024-12-11

**Authors:** Melissa W. Y. Leung, Marloes T. Bazelier, Bernard M. J. Uitdehaag, Hilda J. I. De Jong, Olaf H. Klungel, Ewoudt M. W. van de Garde

**Affiliations:** ^1^ Division of Pharmacoepidemiology and Clinical Pharmacology, Department of Pharmaceutical Sciences, Faculty of Science Utrecht Institute for Pharmaceutical Sciences (UIPS), Utrecht University Utrecht The Netherlands; ^2^ Department of Neurology, MS Center Amsterdam, Amsterdam Neuroscience Amsterdam University Medical Center Amsterdam The Netherlands; ^3^ PHARMO Institute Utrecht The Netherlands; ^4^ Department of Pharmacy Sint Antonius Hospital Utrecht/Nieuwegein The Netherlands

**Keywords:** antibiotics, drug utilization study, infections, multiple sclerosis, Netherlands, nitrofurantoin, urinary tract infection

## Abstract

**Purpose:**

The purpose of this study was to describe the intensity and patterns of antibiotic drug use among people with multiple sclerosis (pwMS) in the Netherlands.

**Methods:**

People with prevalent MS between 1 January 2018 and 31 December 2020 were identified using ambulatory hospital records from the PHARMO Database Network that contains routinely collected healthcare data from the Netherlands. Out‐patient pharmacy dispensing data were used to assess type of antibiotic, dosage, and amounts dispensed. Antibiotic intensity in defined daily doses (DDD)/1000 patient‐days (PD) was calculated together with frequency of dispensing of the same (prolongation) or different (switch) antibiotic up to 3 days after the end of the last antibiotic prescription; and stratified by sex, age, polypharmacy (use of > 4 out‐patient prescription drugs for > 29 days), type of disease‐modifying treatment, and Sars‐CoV‐2‐related lockdown.

**Results:**

A total of 1960 (37.8%) out of 5179 pwMS were dispensed ≥ 1 antibiotic. Of the 8762 dispensing events, 27.6%% were part of a prolongation, and 16.3% of a switch. Overall antibiotic use among pwMS was 18.8 DDD/1000 PD (95% confidence interval [95% CI]: 18.7–19.0) compared to 7.77–8.90 DDD/1000 PD in the general out‐patient population, as reported by the Dutch Working Party on Antibiotic Policy. Antibiotic use was higher among women, increased with age, and was higher in people with polypharmacy and lower during lockdown. Nitrofurantoin was the most commonly dispensed antibiotic (41.7%).

**Conclusions:**

The intensity of antibiotic use is considerably higher among pwMS than the general population. This reflects the burden of infection in this susceptible population.

**Summary**

- This drug utilization study used routinely collected healthcare data from the Netherlands to describe the intensity and patterns of antibiotic drug use among people with multiple sclerosis (MS).
- Antibiotic use was higher among people with MS than the general population; and higher among women, older people, and people with polypharmacy (use of > 4 out‐patient drugs for > 29 days).
- Nitrofurantoin was the antibiotic that was most commonly dispensed (41.7% of all antibiotic dispensing events) and used pre‐switch (44.0% of switches).
- The high intensity of antibiotic use among people with MS reflects the burden of infection in this susceptible population.

## 1 Introduction

People with multiple sclerosis (pwMS) have a higher risk of infection than the general population and fill more antibiotic prescriptions than people without multiple sclerosis (MS) [1, 2]. The risk of infection may be increased by the use of disease‐modifying treatments (DMTs), because their mechanism of MS disease control is through immunomodulation. In the Netherlands, DMTs are categorized into first‐, second‐, and third‐line treatments [3]. The second‐line (fingolimod, cladribine, natalizumab, and ocrelizumab) and third‐line (alemtuzumab) treatments have higher efficacy, but also a higher risk of adverse events including infections [4]. The risk of infection differs between DMTs: a recent systematic review with network meta‐analysis of trial data found that the biggest difference in probability of any infection between two DMTs was nearly 15% (47.2% for ocrelizumab and 62.0% for cladribine), and reported no significant increase between all DMTs and placebo [5].

Out‐patient antibiotic prolongations and switches have been studied in the Netherlands [6] as part of the longstanding history of nationwide antibiotic use surveillance. However, little is known about the pattern of antibiotic use specifically among pwMS in terms of the types of antibiotics used, for how long and how frequently antibiotic switches occur. This is relevant, because it can give insight into the infection burden and management among pwMS. For example, prolonged antibiotic use may reflect complications such as incomplete pathogen clearance and antibiotic switches can proxy a bug‐drug mismatch with standard treatments. Moreover, prolonged infection duration may increase the risk of MS relapse or pseudo‐relapse [7].

Therefore, the objective of this study was to describe the intensity and pattern of antibiotic use among the population with prevalent MS in the Netherlands, and whether intensity of antibiotic drug use is associated with sex, age, polypharmacy of out‐patient drugs, use of second‐ or third‐line DMT, and lockdown implementation to curb the spread of Sars‐CoV‐2.

## 2 Methods

### 2.1 Data Source

Routinely collected healthcare data from the Netherlands were used from the PHARMO Database Network, which is representative of the Dutch population in terms of age and gender, and contains datasets with linkage through deterministic and probabilistic methods [8]. Three datasets were used: the ambulatory contacts dataset, add‐on pharmacy dataset, and out‐patient pharmacy dataset. The ambulatory contacts dataset contains information on out‐patient consultation dates and diagnoses from approximately 80% of the hospitals in the Netherlands, and is International Classification of Disease (ICD)‐coded. The add‐on pharmacy dataset contains information on high‐budget impact medication and covers approximately 80% of the hospitals in the Netherlands. The out‐patient pharmacy dataset contains information on dispensing events of prescription drugs from community pharmacies and covers approximately 25% of the Dutch population who have ever collected ≥ 1 drug. The add‐on and out‐patient pharmacy datasets are both Anatomical Therapeutic Chemical (ATC)‐coded.

### 2.2 Study Design and Population

This was a cross‐sectional drug utilization study of antibiotics among pwMS. The study population was selected based on presence of at least two ambulatory records of MS (ICD‐10 code G35) anytime between 1 January 2016 and 31 December 2020, of which at least one recorded on or after 1 January 2018. In addition, out‐patient pharmacy data was required for at least 30 days of the study period (i.e., 1 January 2018–31 December 2020). People younger than 18 years old at the first ambulatory record of MS during the study period were excluded. Follow‐up for antibiotic use of pwMS who met the inclusion criteria of ≥ 2 ambulatory records of MS and out‐patient pharmacy data availability started on the index date: either the date of the first available record of ambulatory contact for MS between 1 January 2018 and 31 December 2020 or start date of data availability in the out‐patient pharmacy database during the study period (whichever occurred last). Follow‐up ended on date of death, deregistration from the out‐patient pharmacy dataset, or end of the study period (whichever occurred first).

### 2.3 Drug Utilization of Out‐Patient Antibiotics

Antibiotic drug use was ascertained from out‐patient pharmacy dispensing records of all antibiotic drugs for systemic use (ATC code starting with J01) [6]. The amount of antibiotic use was assessed by defined daily dose (DDD) and standardized as intensity in DDD per 1000 person‐days (PD). The types of antibiotic drugs used were studied on the level of the name of the active pharmaceutical substance. Antibiotic use windows were constructed, starting on the dispensing date and ending on the dispensing date plus duration of use in days, to study antibiotic prolongations and switches. Prolongations were defined as repeated dispensing events of the same antibiotic drug during an antibiotic use window or up to 3 days after the end of the last antibiotic use window [6]. Switches were defined as the dispensing of a different antibiotic drug during the last antibiotic use window or up to 3 days after the end of the last antibiotic use window [6]. Each antibiotic dispensing event was categorized as a single‐dispensing antibiotic use window, antibiotic prolongation, or switch.

### 2.4 Covariates

Sex, age, out‐patient prescription drug use, second‐ and third‐line DMT use, and presence of lockdown to curb the spread of the Sars‐CoV‐2 virus were determined at index date. Polypharmacy was defined as simultaneous use of > 4 different (at the third level of ATC code) prescription drugs with an ATC code recorded in the out‐patient pharmacy dataset for > 29 days, covering the index date [9]. Second‐ and third‐line DMT use was assessed using the 6‐month history in the out‐patient pharmacy and add‐on datasets. The second‐ and third‐line DMTs included in this study were alemtuzumab, natalizumab, ocrelizumab, and fingolimod (ATC code list is available as Supporting Information Material). Cladribine use was not ascertained in this study because cladribine is dispensed by an external organization and therefore its use was not recorded in our data. DMT use windows were constructed starting on the dispensing date and ending after a fixed duration: prescription duration +42 days for fingolimod (based on daily administration and 6‐week clearance) [10, 11]; 90 days for natalizumab (based on administration every 4 weeks and 2–3 month elimination) [10, 12]; 180 days for ocrelizumab (based on administration every 6 months) [13]; and 365 days for alemtuzumab (based on one administration per year) [14]. If a new DMT was prescribed during an ongoing DMT use window, the current DMT was cut off for an immediate switch to the new DMT. Follow‐up time outside the DMT use windows was classified as no use. For the stratification by presence of lockdown to curb the spread of the Sars‐CoV‐2 virus, follow‐up time from 15 March 2020 until 1 June 2020 and from 14 October 2020 until 31 December 2020 was categorized as during lockdown [15].

### 2.5 Statistical Analysis

#### 2.5.1 Descriptive Statistics

The median age with corresponding interquartile range (IQR) of the study population was calculated, as well as percentages of the study population per category of sex, second‐ or third‐line DMT use, and number of out‐patient prescription drugs used.

#### 2.5.2 Drug Utilization of Out‐Patient Antibiotics

The mean and 95% confidence interval (CI) of number of DDDs of out‐patient antibiotic (DDD)/1000 PD was calculated overall and by sex, age, polypharmacy of out‐patient prescription drugs, use of second‐ or third‐line DMT, and presence of lockdown to curb the spread of the Sars‐CoV‐2 virus. The types of antibiotic drugs used were assessed by the total number of dispensing events of each antibiotic drug (active pharmaceutical substance) and its percentage of the total number of antibiotic dispensing events. The number of antibiotic dispensing events that were part of antibiotic prolongation or switch were summed and expressed as a percentage of the total number of antibiotic dispensing events. Also, the switches from the first to the second antibiotic drug were visualized in a Sankey diagram. All analyses were conducted using R statistical software (version 4.1.1).

#### 2.5.3 Sensitivity analysis

A sensitivity analysis was conducted to remove any effects of long‐term antibiotic use on the findings. Long‐term use was defined as three or more consecutive prescriptions of the same antibiotic (i.e., repeated dispensing events of the same antibiotic drug during an antibiotic use window or up to 3 days after the end of the last antibiotic use window) or continuous use of over 56 days. The statistical analyses for types of antibiotic drugs, and antibiotic prolongations and switches were repeated excluding prescriptions for long‐term antibiotic use.

## 3 Results

### 3.1 Study Population

**FIGURE 1:**
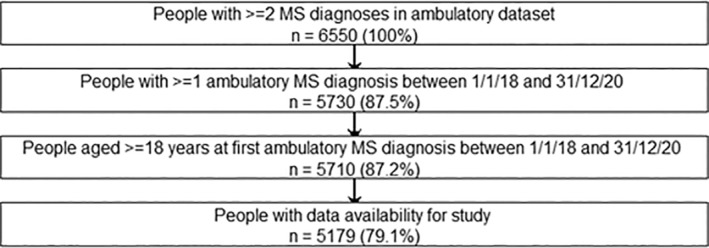
Flowchart of inclusion of the study population.

A total of 6550 people had at least two ambulatory records of MS between 1 January 2016 and 31 December 2020, of whom 5179 (79.1%) met all eligibility criteria (Figure 1). A total of 3797 (73.3%) were women and the median [IQR] age at start of follow‐up was 49 [39–58] years (Table 1). A total of 4896 (94.0%) people had no record of a DMT at start of follow‐up, and 702 (13.6%) had polypharmacy. The total follow‐up time was 11 129 person‐years (PY). The median [IQR] follow‐up time was 2.40 [1.72–2.78] years. Fewer than 10 people used alemtuzumab during follow‐up.

**TABLE 1:** Characteristics of the study population at the start of follow‐up.

	People with MS (*n* = 5179)
Women, *n* (%)	3797 (73.3)
Age, years [interquartile range]	49 [39–58]
Second‐ or third‐line DMT use at start of follow‐up, *n* (%)	
No or unknown^a^	4869 (94.0)
Fingolimod	172 (3.3)
Natalizumab	128 (2.5)
Ocrelizumab	10 (0.2)
Number of out‐patient drugs used, *n* (%)	
0	2222 (42.9)
1–2	1558 (30.1)
3–4	697 (13.5)
5–6	372 (7.2)
7–8	183 (3.5)
9–10	86 (1.7)
> 10	61 (1.2)

^a^ No DMT use ascertained from the available data.

### 3.2 Drug Utilization of Out‐Patient Antibiotics

#### 3.2.1 Intensity

A total of 1960 (37.8%) people used at least one antibiotic during follow‐up. The total number of antibiotic dispensing events was 8762. Mean [95% CI] antibiotic use overall was 18.8 [18.7–19.0] DDD/1000 PD. Intensity of antibiotic use was higher among women (19.9 [19.8–20.1] DDD/1000 PD) than men (15.9 [15.6–16.1] DDD/1000 PD), increased with age (from 9.9 [9.8–10.1] DDD/1000 PD among people aged 18–40 years at start of follow‐up to 29.2 [28.8–29.5] DDD/1000 PD among people aged 61 years or over at start of follow‐up), and was higher among people with polypharmacy (56.8 [56.2–57.4] DDD/1000 PD) than people not with polypharmacy (12.7 [12.6–12.8] DDD/1000 PD) (Table 2). Intensity of antibiotic use during second‐ or third‐line DMT use (19.5 [19.0–19.9] DDD/1000 PD) was similar to that during no or unknown use (18.8 [18.6–18.9] DDD/1000 PD), and higher outside lockdown (19.1 [18.9–19.2] DDD/1000 PD) to curb the spread of the Sars‐CoV‐2 virus than during (17.6 [17.3–17.9] DDD/1000 PD).

**TABLE 2:** Antibiotic use by sex, age at index date, polypharmacy status at index date, disease‐modifying treatment (DMT) exposure, and Sars‐CoV‐2‐related lockdown implementation status.

	People with ≥ 1AB dispensed/people at risk (%)	ABs dispensed (*n*)	FU time, years	AB DDD/1000 person‐days [95% CI]
Total	1960/5179 (37.8%)	8762	11 129.0	18.8 [18.7–19.0]
Sex				
Women	1546/3797 (40.7%)	7016	8172.7	19.9 [19.8–20.1]
Men	414/1382 (30%)	1746	2956.3	15.9 [15.6–16.1]
Age (years) at the start of follow‐up				
18–40	465/1497 (31.1%)	1466	3144.7	9.9 [9.8–10.1]
41–50	463/1301 (35.6%)	2088	2840.1	17.0 [16.8–17.3]
51–60	567/1358 (41.8%)	2683	2983.9	22.5 [22.2–22.8]
61+	465/1023 (45.5%)	2525	2160.4	29.2 [28.8–29.5]
Polypharmacy^a^ at the start of follow‐up				
No	1497/4477 (33.4%)	5265	9569.1	12.7 [12.6–12.8]
Yes	463/702 (66%)	3497	1559.9	56.8 [56.2–57.4]
Second‐ or third‐line DMT use				
Yes	235/649 (36.2%)	819	1047.9	19.5 [19.0–19.9]
Fingolimod	82/234 (35%)	273	393.5	14.5 [13.9–15.1]
Natalizumab	80/201 (39.8%)	336	345.6	25.1 [24.3–26.0]
Ocrelizumab	84/270 (31.1%)	209	307.7	19.6 [18.8–20.4]
No or unknown^b^	1780/4987 (35.7%)	7943	10 081.1	18.8 [18.6–18.9]
Lockdown to curb the spread of Sars‐CoV‐2				
No	1815/5124 (35.4%)	7540	9342.5	19.1 [18.9–19.2]
Yes	612/4511 (13.6%)	1222	1786.5	17.6 [17.3–17.9]

*Note:* No results reported for alemtuzumab use because of limited total FU time on alemtuzumab (total 1.1 person‐year).

Abbreviations: AB: Antibiotic; DDD: Defined daily doses; FU: Follow‐up; CI: Confidence interval; PD: Person‐days.

^a^ Use of > 4 out‐patient drugs for > 29 days.

^b^ No DMT use ascertained in the available data.

#### 3.2.2 Types of Antibiotic Drugs

Nitrofurantoin was the antibiotic that accounted for the highest percentage of antibiotic dispensing events: 41.7%. The second most commonly dispensed antibiotic was fosfomycin (9.0%). The 10 most commonly dispensed antibiotics together accounted for 92.8% of all antibiotic dispensing events (Table 3).

**TABLE 3:** Distribution of dispensing events across the ten most commonly dispensed antibiotics (*n* = 8762 total dispensing events).

Antibiotic	Percentage (n)
Nitrofurantoin	41.7% (3655)
Fosfomycin	9.0% (790)
Ciprofloxacin	5.5% (773)
Amoxicillin	8.3% (726)
Amoxicillin/clavulanic acid	7.0% (609)
Trimethoprim	5.8% (510)
Doxycycline	4.0% (352)
Cotrimoxazole	3.2% (282)
Flucloxacillin	2.8% (247)
Azithromycin	2.2% (190)

#### 3.2.3 Antibiotic Prolongations and Switches

**FIGURE 2:**
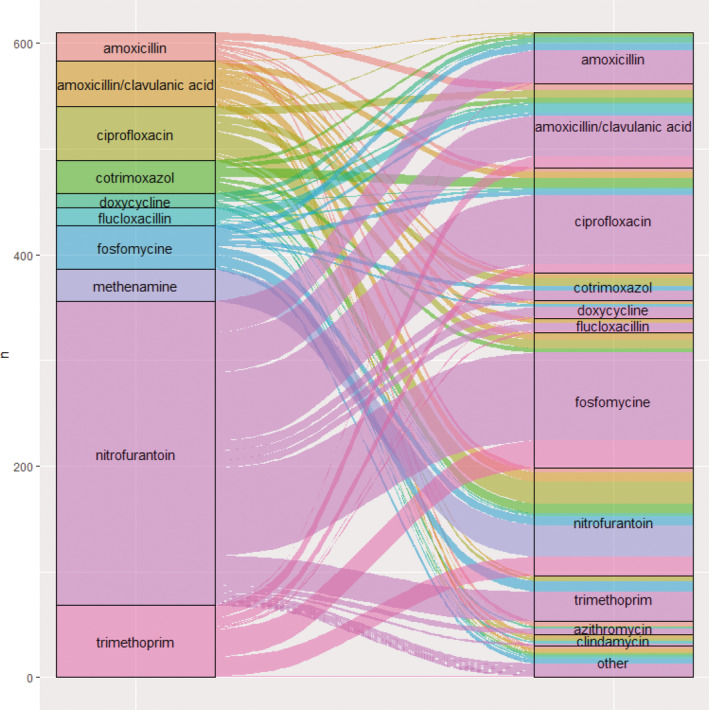
Number of switches to a second antibiotic from the 10 most commonly used antibiotics up to the first switch. “Other” antibiotics were: Benzathinebenzylpenicilline, cefuroxime, clarithromycin, feneticillin, gentamicin, levofloxacin, linezolid, methenamine, minocycline, moxifloxacin, and norfloxacine.

The total of 8762 antibiotic dispensing events comprised 4912 single dispensing events, 2420 (27.6%) dispensing events that were part of antibiotic prolongation and 1430 (16.3%) of switch. The 10 most frequently used antibiotics up to the first antibiotic switch accounted for 610 (93.1%) of the switches from the first to second antibiotic (Figure 2). A total of 288 (44.0%) switches were from nitrofurantoin.

### 3.3 Sensitivity Analysis

A total of 66.5% (*n* = 5826) of all dispensing events were included in the sensitivity analysis in which long‐term antibiotic use was excluded. The percentage of dispensing events for nitrofurantoin was 28.9% (1684 events), compared with 41.7% in the main analysis. The percentages of ciprofloxacin, amoxicillin, fosfomycin, and amoxicillin/clavulanic acid were slightly higher than in the main analysis (Table S1). A lower percentage of dispensing events was part of antibiotic prolongation: 4.8% (277 dispensing events) compared with 27.6% (2420 dispensing events) in the main analysis. Results of antibiotic switches were similar in the sensitivity analysis to the main analysis (Data S1).

## 4 Discussion

Overall, 37.8% of the cohort of people with prevalent MS in the Netherlands used at least one antibiotic during a follow‐up time of up to 3 years (median [IQR]: 2.40 [1.72–2.78]), with mean [95% CI] intensity of antibiotic use 18.8 [18.7–19.0] DDD/1000 PD. People with female sex, higher age, and polypharmacy of out‐patient drugs had higher intensity of antibiotic drug use. Nitrofurantoin and fosfomycin both used primarily to treat UTI, together accounted for approximately half of all antibiotic dispensing events. A total of 27.6% of dispensing events were part of antibiotic prolongation and 16.3% of switch. 44.0% of switches were from nitrofurantoin.

The intensity of antibiotic use among pwMS in the Netherlands of 18.8 DDD/1000 PD was higher than among the general population: total outpatient use of antibiotics among the general population was 8.90 DDD/1000 inhabitant‐days in 2018, decreasing to 7.77 DDD/1000 inhabitant days in 2020, and increasing to 8.32 DDD/1000 inhabitant‐days in 2022 [16, 17, 18, 19]. The decrease in 2020 was reflected in our study in the stratified results on antibiotic use intensity by presence or absence of lockdown to curb the spread of the Sars‐CoV‐2 virus. In a recent retrospective study assessing the risk of outpatient infections in a US cohort [20], women with MS had a higher risk, as was the case in our study. However, in that study, increased age was associated with a slight decrease in risk of infection, while we observed an increase in the intensity of antibiotic use with increasing age. The US study also found associations with DMT use, which we could not study because of limited follow‐up time on second‐ or third line DMTs and because first‐line DMT use could not be ascertained. Lastly, we observed higher antibiotic drug use intensity among people with polypharmacy of out‐patient drugs as a proxy for overall health, while in the US study comorbidity and diabetes were not identified as outpatient risk factors (although COPD and obesity were). This may be due to differences between polypharmacy and comorbidities as proxies for overall health.

The overall prevalence of antibiotic use of 37.8% of pwMS over a period of up to 3 years was similar to the prevalence in a study in the United States (July 2015–December 2018) [21], in which 36.8%–38.7% of pwMS and with DMT use experienced an infection, of whom 84.3%–86.5% also had an antimicrobial prescription. A study on out‐patient antibiotic switching in the Netherlands between 2006 and 2014 [6] (excluding long‐term [i.e., > 14‐day] prescriptions and prescriptions from people with annual antibiotic exposure > 8 weeks) found that 2% of antibiotic dispensing events were part of a switch, which is lower than the 14.4% found in our sensitivity analysis excluding long‐term antibiotic use. The higher antibiotic switch prevalence in our study could reflect that antibiotic treatment failure is more probable among pwMS than the general population. Similar to our study, nitrofurantoin was the most commonly first used antibiotic in switches, accounting for 27% of switches from first to second antibiotic (41% in our sensitivity analysis excluding long‐term antibiotic use). As in our study, the most common post‐switch antibiotics (not “other”) included ciprofloxacin, fosfomycin, amoxicillin, and amoxicillin/clavulanic acid; but also azithromycin and clarithromycin rather than nitrofurantoin and trimethoprim.

As a confirmation of the known increased risk of infection among pwMS compared with the general population, our study underscores the importance of infection risk management in this susceptible population. Conversely, antibiotic use intensity could also be considered as a proxy for DMT effectiveness, as less control over the MS disease process may result in an increased frequency of UTI and thus need for antibiotics. In this scenario, follow‐up of antibiotic usage could act as sentinel for bladder dysfunction, which is known to be a patient‐relevant outcome in MS [22]. The large share of antibiotics used to treat UTI and the frequency of switches between them suggest that the burden of UTI is a large share of the overall infection burden on pwMS. Although the relatively high switch percentage from nitrofurantoin (14% vs. 5% in the general population) [6] could suggest that nitrofurantoin is not optimal as empirical treatment in pwMS, who are known to experience UTIs frequently, it still reflects adherence to the Standard for Urinary Tract Infections of the Dutch College of General Practitioners: the Standard recommends nitrofurantoin as the first‐choice antibiotic and urinary culture for people from an at‐risk group [23]. Most switches were to broader‐spectrum antibiotics such as amoxicillin‐clavulanic acid, ciprofloxacin, and fosfomycin. This suggests that the spectrum of nitrofurantoin is too narrow to achieve at least 90% probability of target attainment in this specific population. A next step toward deciding on any potential alternative empirical antibiotic would be follow‐up research including the detection of pathogens and susceptibilities in this population. We could not do this, because indications and microbiology results were not available in our datasets.

Strength of this study was that the study population covered approximately 20% of the population with MS in the Netherlands [24]. The 20% coverage is in line with the 25% coverage of the Dutch population who has ever collected an out‐patient prescription drug, and linkage with the other datasets, which cover 80% of Dutch hospitals. Misclassification of MS or antibiotic dispensing is not expected in this study, so any effect of selection bias on the results is expected to be limited. In addition to describing the intensity of antibiotic use among pwMS, we could compare the intensity of antibiotic use among pwMS to that among the general population in the Netherlands, given the comprehensive knowledge of nationwide antibiotic use in the Netherlands [25]. The main limitation was that the out‐patient pharmacy dataset did not include diagnosis codes. Data on the indication for out‐patient antibiotic treatment, reason for switching, and medical complexity of the study population associated with out‐patient drug use were thus missing. These could be studied further to provide more certainty of which infections are most likely to require an antibiotic switch, whether the switch can be avoided, and which pwMS are most at risk given their comorbidity profile. In addition, we could not ascertain use of first‐line DMTs because they are delivered through external organizations in the Netherlands. As a result, we could only partly study DMT use in relation to antibiotic use. Also, the findings on the intensity of antibiotic use were not compared to age‐ or gender‐matched data from the general population. Lastly, in‐patient data on antibiotic treatment were not included in this study, which means that severe infections could not be studied and prolongations and switches may have been under‐ascertained. This suggests that the true frequency of antibiotic prolongations and switches could be higher than reported here.

The high intensity of antibiotic use among pwMS reflects the burden of infection in a susceptible population. UTIs appear a particular challenge in the infection management among pwMS. This study highlights the importance of infection risk management among pwMS, and especially for women, older people, and people with polypharmacy.

### 4.1 Plain Language Summary

MS is a chronic condition in which a person's immune system damages the protective layer around cells in the brain and spinal cord. People with MS (pwMS) are more prone to infection than the general population. The aim of our study was to describe the use of antibiotics among pwMS in the Netherlands, for which we used anonymized hospital and pharmacy data. We found that pwMS used more antibiotics than the general population. Antibiotic use was more frequent among women and people who used > 4 out‐patient drugs for > 29 days, and increased with age. Nitrofurantoin, the first‐choice antibiotic to treat UTIs, was the most commonly dispensed antibiotic (41.7% of all antibiotic dispensing events). The high intensity of antibiotic use among pwMS reflects the burden of infection in this susceptible population and highlights the importance of appropriate infection risk management among pwMS.

## Ethics Statement

This study was approved by the Compliance Committee, the independent privacy and governance board that controls the use of study‐specific datasets from the PHARMO Database Network. This study used only anonymized data from the PHARMO Database Network and was therefore not subject to ethics review.

## Conflicts of Interest

BU has received consultancy fees from Immunic Therapeutics. The other authors declare no conflicts of interest.

## Supporting information


**Data S1.** Supporting Information.

## Data Availability

Code lists and results from the sensitivity analysis are available as Supporting Information Material.
