# Supplementary material for: Antibiotic Utilization Among People With Multiple Sclerosis in the Netherlands, 2018–2020
Source: Pharmacoepidemiol Drug Saf. 2024 Dec 11;33(12):e70070. doi: 10.1002/pds.70070 (PMC11634561; doi:10.1002/pds.70070)
Supplement: Supplementary file 1 — Data S1. Supporting Information. [file PDS-33-e70070-s001.zip › pds-24-0392-File005.docx]

## Outpatient antibiotic use

Most commonly dispensed antibiotics

Out of 5826 dispensing events excluding long-term antibiotic use, 28.9% (n = 1684) were for nitrofurantoin. The next most commonly dispensed antibiotic was ciprofloxacin (12.2%). The ten most commonly dispensed antibiotics accounted for 94.5% of the short-term antibiotic dispensing events (Table S1).

**Table S1** Distribution of dispensing events across the ten most commonly dispensed antibiotics excluding long-term antibiotic use (n = 5826 total dispensing events)

**Antibiotic Percentage (n)**

nitrofurantoin 28.9% (1684)

ciprofloxacin 12.2% (713)

amoxicillin 12.0% (702)

fosfomycine 11.8% (687)

amoxicillin/clavulanic acid 9.9% (574)

doxycycline 5.5% (319)

flucloxacillin 4.1% (240)

trimethoprim 4.0% (232)

cotrimoxazol 3.1% (182)

azithromycin 3.0% (172)

Antibiotic prolongations and switches

The total 5826 antibiotic dispensing events excluding long-term antibiotic use comprised 4710 single dispensing events; 277 (4.8%) dispensing events were part of antibiotic prolongation and 839 (14.4%) of switch. The nine most frequently used antibiotics up to the first antibiotic switch of an antibiotic use window accounted for 362 (93.3%) of the switches from the first to second antibiotic (Figure 2). 159 (41%) switches were from nitrofurantoin, with the majority to a second-line treatment for UTI (i.e., amoxicillin/clavulanic acid, ciprofloxacin, fosfomycin, or trimethoprim).


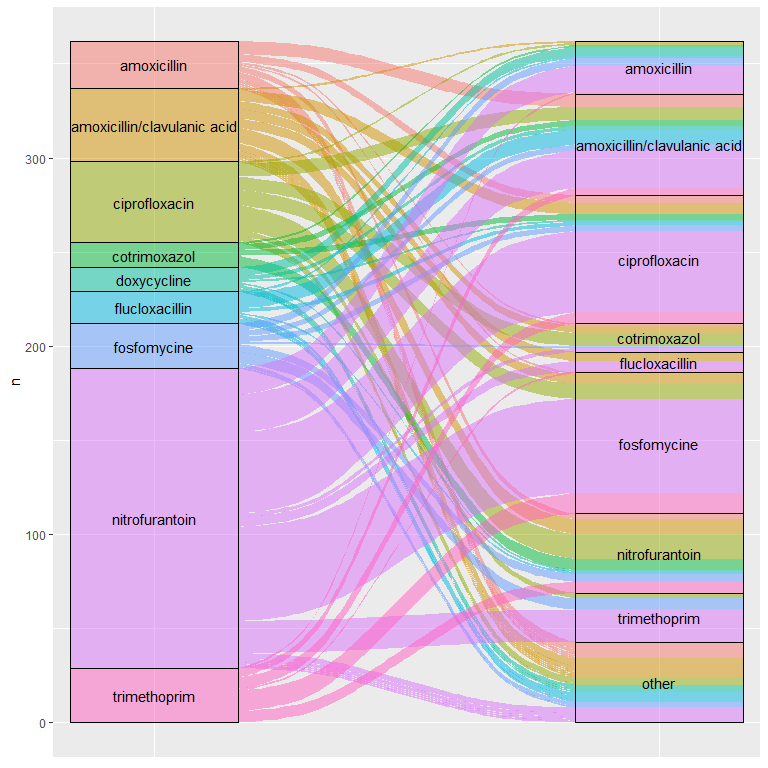


**Figure S1** Number of people switching to a second antibiotic from the nine most commonly used antibiotics up to the first switch excluding long-term antibiotic use. ‘Other’ antibiotics were: azithromycin, cefuroxime, clarithromycin, clindamycin, doxycycline, gentamicin, levofloxacin, moxifloxacin, and norfloxacine.
